# Supplementary material for: Inhibitory neurons in the superior colliculus mediate selection of spatially-directed movements
Source: Commun Biol. 2021 Jun 11;4:719. doi: 10.1038/s42003-021-02248-1 (PMC8196039; doi:10.1038/s42003-021-02248-1)
Supplement: Supplementary file 2 — Supplementary Information [file 42003_2021_2248_MOESM2_ESM.pdf]

Inhibitory neurons in the superior colliculus mediate selection of spatially-directed movements

Jaclyn Essig, Joshua B. Hunt, and Gidon Felsen

Supplementary Figures and Tables

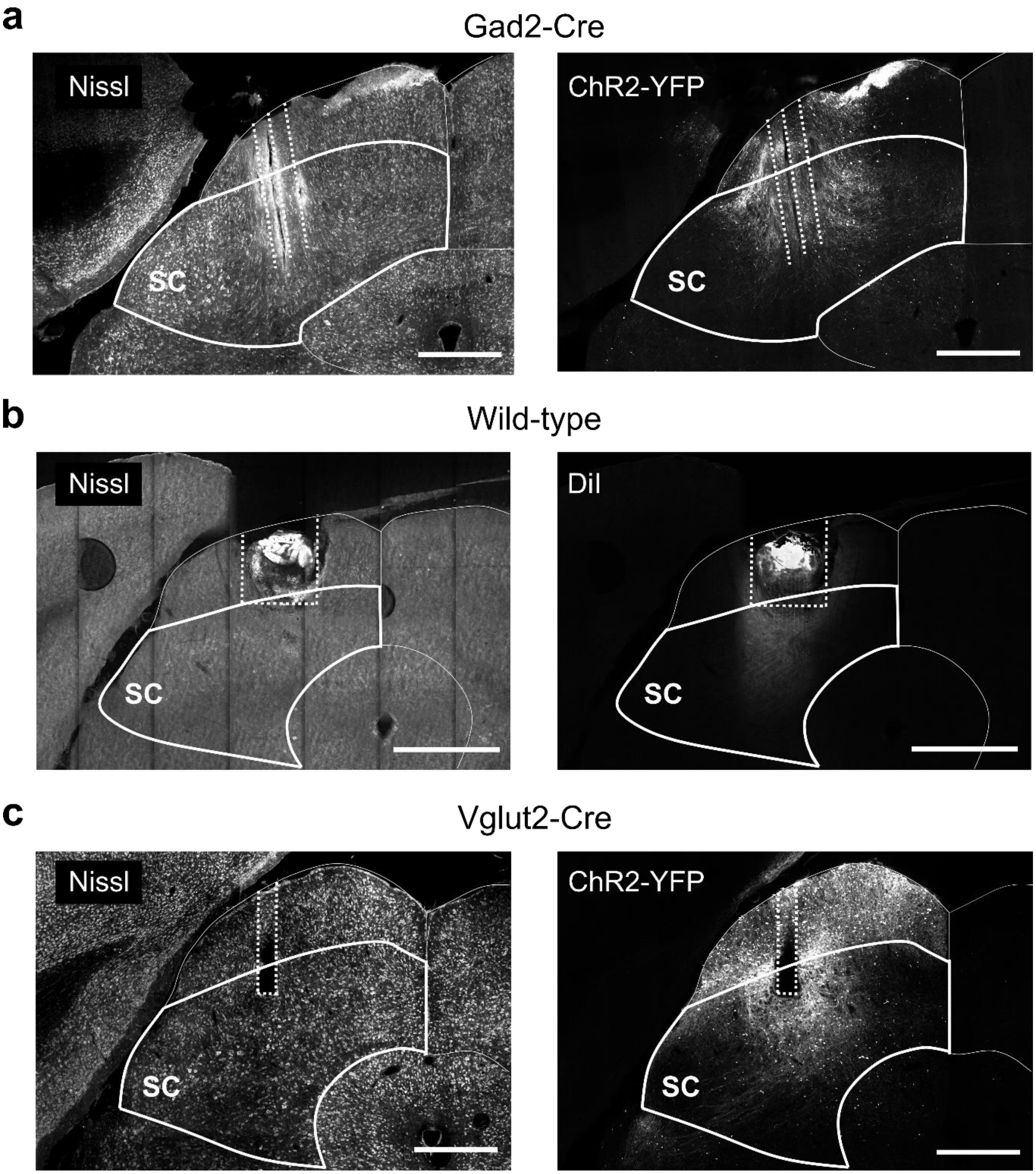

Supplementary Figure 1: Representative coronal histological sections showing ChR2-YFP expression and targeting of intermediate layer of SC.
(**a**) Nissl and ChR2-YFP expression in left SC of Gad2-Cre mouse. Dashed lines show visible optetrode tracks. Scale bar, 500 µm.
(**b**) Nissl expression and DiI in left SC of wild-type mouse used in muscimol experiments. DiI coated the internal cannula used for drug delivery. Dashed lines show visible guide cannula track. Scale bar, 500 µm.
(**c**) Nissl and ChR2-YFP expression in left SC of Vglut2-Cre mouse. Dashed lines show visible optical fiber track. Scale bar, 500 µm.

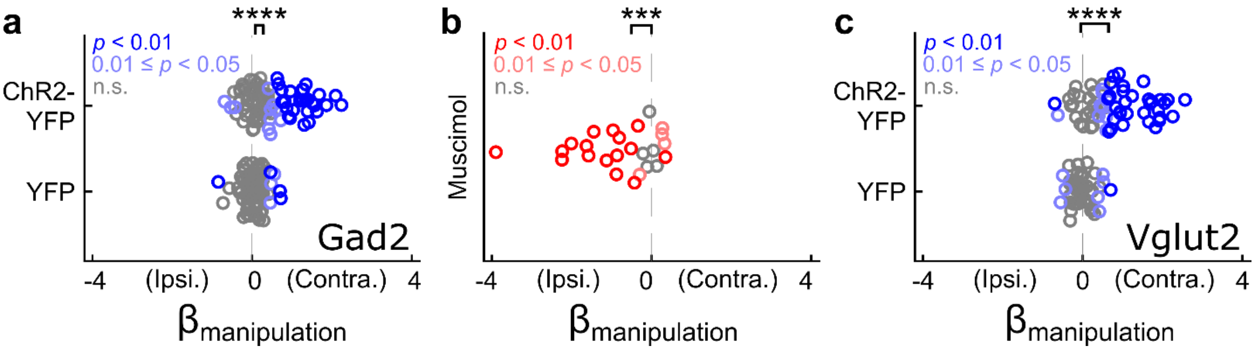

Supplementary Figure 2: Optogenetic control and muscimol results by session.
(**a**) Effect of GABAergic photoactivation (ChR2-YFP; n = 96) compared to control (YFP; n = 63) sessions (**** $p = 8.19 \times 10^{-5}$, $U = 3921$, two-tailed Mann-Whitney U test). Bracket, difference between group medians (ChR2-YFP = 0.28; YFP = 0.08).
(**b**) Effect of muscimol across sessions (n = 25; *** $p = 0.00089$, W = 39, two-tailed Wilcoxon signed-rank test). Bracket, group median (= -0.51) from zero.
(**c**) As in **a**, for glutamatergic photoactivation (ChR2-YFP; n = 64) compared to control (YFP; n = 52) sessions (**** $p = 7.1 \times 10^{-10}$, $U = 1931$, two-tailed Mann-Whitney U test). Bracket, difference between group medians (ChR2-YFP = 0.65; YFP = -0.05).

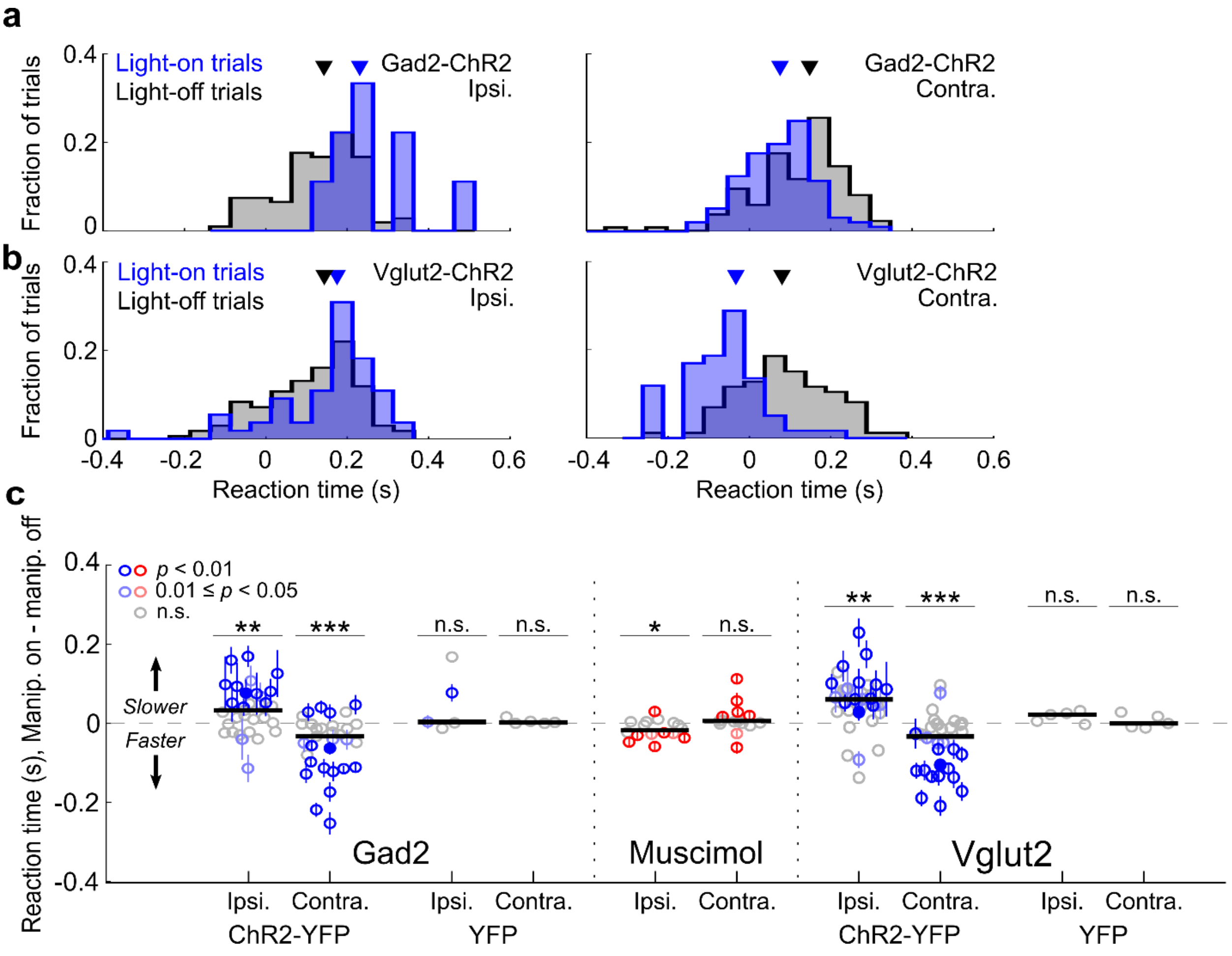

Supplementary Figure 3: Effect of manipulating SC activity on reaction times.
(**a**) Distributions of reaction times to the go signal (from go signal to odor port exit) for light-on and light-off trials, separately for ipsilateral (left panel) and contralateral (right panel) choices, for an example session in which GABAergic neurons were photoactivated. Trials with negative reaction times indicate that the mouse exited the odor port before the go signal; these trials were not rewarded but are included in behavioral analyses if a choice was made. Black arrowhead, median light-off reaction time; blue arrowhead, median light-on reaction time.
(**b**) As in **a**, for an example session in which glutamatergic neurons were photoactivated.
(**c**) For each session in which $\beta_{manipulation}$ exhibited the dominant effect ($p < 0.05$) for the given experiment (e.g., $\beta_{manipulation} > 0$ for photoactivation of GABAergic neurons; Fig. 2b), median reaction time was calculated separately for light-on and light-off ipsilateral and contralateral choices (as in **a**). The difference in medians (± SEM) within each direction is shown, separately for each group of experiments. Each session is represented by 2 symbols (1 for ipsilateral trials and 1 for contralateral trials). Horizontal lines show median within group for each direction. For muscimol sessions, reaction times were compared to the preceding and following saline sessions. All groups were compared to zero using a two-tailed Wilcoxon signed-rank test (*** $p < 0.001$; ** $p < 0.01$; * $p < 0.05$; n.s., not significant). Filled symbols show example sessions in **a** and **b**.

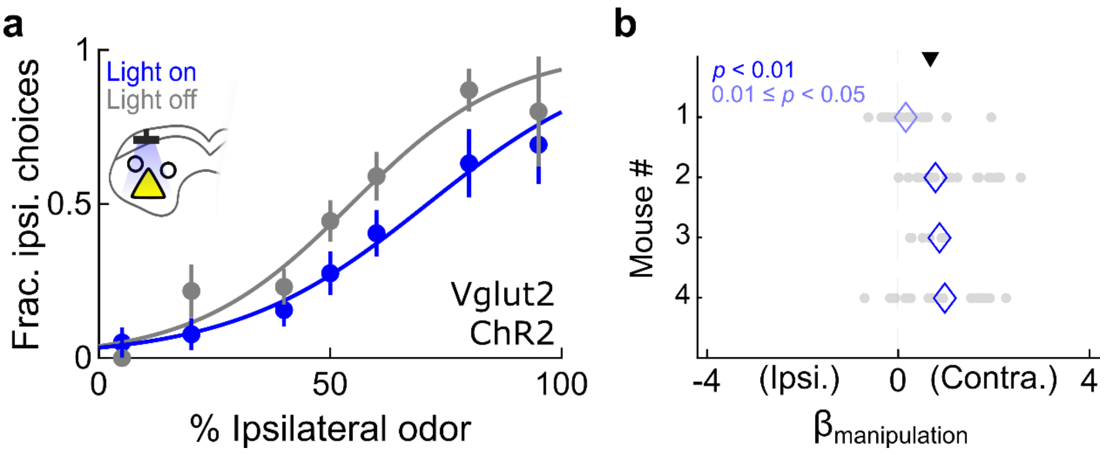

Supplementary Figure 4: Photoactivation of glutamatergic SC neurons during spatial choice promotes contralateral choices.

(**a**) Example session in which glutamatergic neurons were unilaterally photoactivated during spatial choice.

(**b**) Overall influence of glutamatergic photoactivation (n = 4 mice; permutation test) on choice, quantified by logistic regression (Methods). See also Supplementary Fig. 2c for comparison to YFP controls and Supplementary Fig. 3b,c for reaction times. Mice are sorted by effect size. Arrowhead, example session in **a**.

Supplementary Figure 5: Optogenetic identification of GABAergic SC neurons.

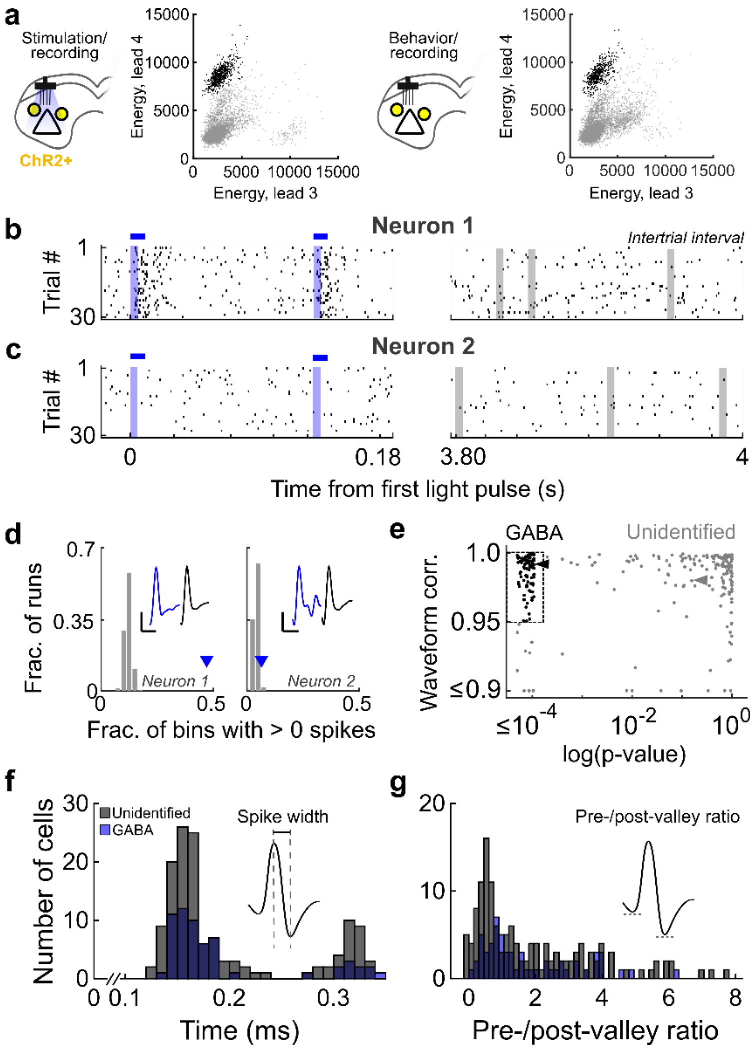

(**a**) Overview: Optotagging session performed (left) in conjunction with behavior (right). Neurons were identified by consistent low-latency responses to light (**b**-**e**) and "tracked" during the behavioral session based on clustering of waveform features. Energy is the square root of the sum of the squared voltage, reflecting the height and depth of the waveform peak and valley, respectively. Each symbol shows 1 spike waveform; black corresponds to waveforms of identified GABAergic neuron.

(**b**) Example rasters for 1 neuron in optotagging session during light-on (left) and light-off periods (intertrial interval, right). Optotagging sessions consisted of at least 50 trials (only 30 trials shown) of 8Hz light delivery (10 ms light-on/115 ms light-off) followed by 4 s of no light delivery. Analysis of light-induced activity was limited to the first 5 ms. Blue shading, 5 ms analysis bins during light delivery. Horizontal blue lines show total duration of light pulses (10ms; only 2 of 10 per trial shown). Gray shading, randomly sampled 5 ms analysis bins during the intertrial interval for 1 run of the simulation (only 3 of 10 per trial shown).

(**c**) As in **b**, for a second example neuron.

(**d**) Left: Gray: For neuron shown in **b**, frequency (across 5000 simulation runs) of the fraction of light-off analysis bins that contain > 0 spikes. Blue arrowhead, fraction of light-on analysis bins that contain > 0 spikes, which differed from light-off distribution ($p < 0.0002$, permutation test). Insets, mean spike waveforms during light-on periods (i.e. light-driven, blue) and light-off periods (i.e., spontaneous, black) from 1 tetrode lead (waveform correlation = 0.992). Scale bars, 25 µV by 500 µs. Right: As described, for neuron shown in **c**. Waveform correlation = 0.976.

(**e**) GABAergic neurons were identified based on a low probability that the measured fraction of light-on analysis bins containing > 0 spikes occurred by chance ($p < 0.0002$, vertical dashed line) and a high correlation between mean spike waveforms during light-on and light-off periods ($r^2 > 0.95$, horizontal dashed line). Each symbol corresponds to 1 neuron (black, identified GABAergic; gray, unidentified). Black arrowhead, neuron shown in **b**; gray arrowhead, neuron shown in **c**.

(**f-g**) Analysis of waveform features: spike width (**f**), and pre-valley to post-valley ratio (**g**). All other waveform features yielded similar results to **g** and **f**.

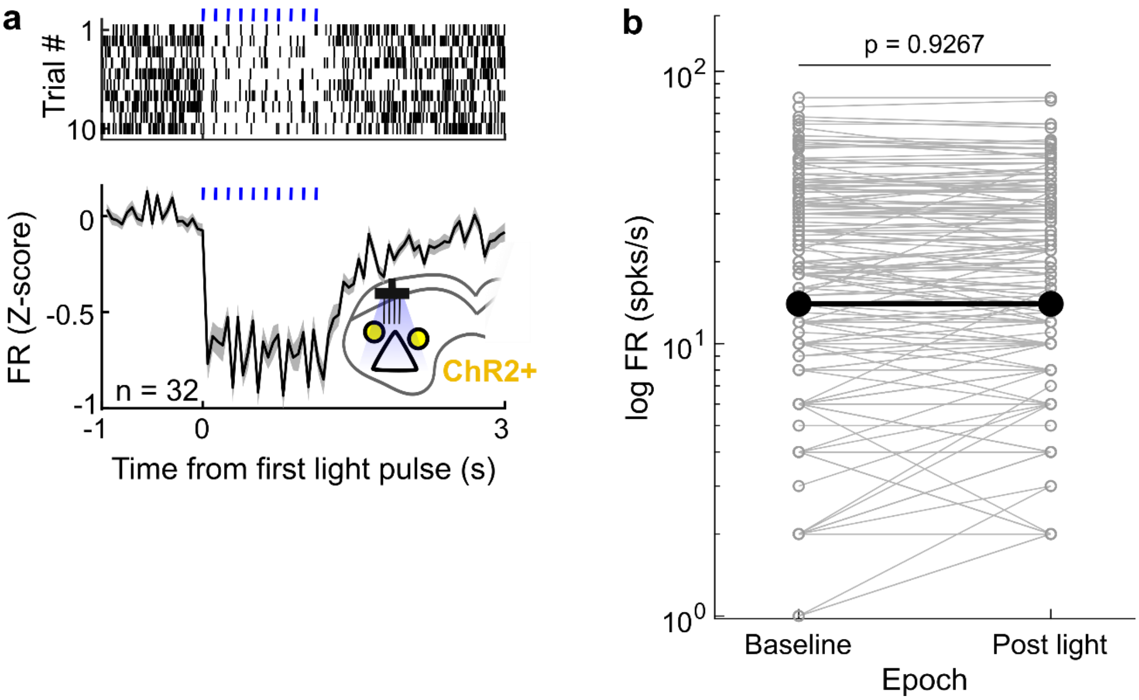

Supplementary Figure 6: Effect of photoactivating GABAergic SC neurons during stimulation/recording sessions on neighboring neurons.

(**a**) Subpopulation of SC neurons inhibited by photoactivation of nearby GABAergic neurons. Top: Raster for example unidentified neuron. Blue ticks, light delivery. Bottom: mean z-scored firing rate across neurons inhibited by light (n = 32/301 neurons (1 GABAergic, 31 unidentified); $p < 0.05$, one-tailed Wilcoxon signed-rank test comparing firing rate during light delivery and intertrial interval). Gray shading, ± SEM.

(**b**) Activity during 500 ms following light delivery did not differ from activity during 500 ms preceding light delivery in unidentified neurons (n = 198; $p = 0.9267$, two-tailed Wilcoxon signed-rank test). Each neuron is represented by a connected pair of small symbols. Large symbols show population medians.

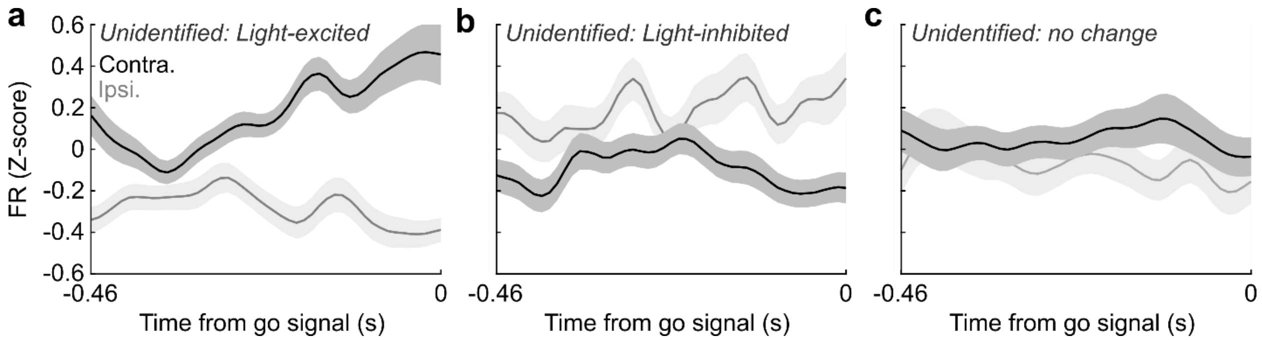

Supplementary Figure 7: Endogenous activity during the choice epoch of unidentified neurons recorded in stimulation/behavior/recording sessions.
(**a**) Mean z-scored firing rate on light-off trials for the subpopulation of unidentified neurons that increase their firing rate during the choice epoch on light-on trials (Light-excited; n = 11/51 neurons).
(**b**) As in **a**, for unidentified neurons that decrease their firing rate during the choice epoch on light-on trials (Light-inhibited; n = 13/51 neurons).
(**c**) As in **a**, for unidentified neurons that do not change their firing rate during the choice epoch on light-on trials (no change; n = 27/51 neurons).

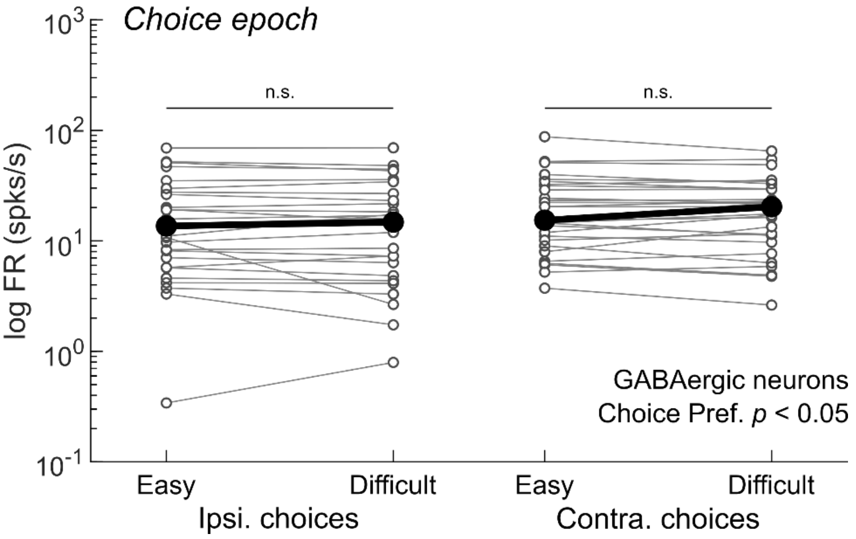

Supplementary Figure 8: Dependence on trial difficulty of endogenous activity of GABAergic SC neurons during spatial choice.
Activity of GABAergic neurons with a significant choice preference ($p < 0.05$; black bars in Fig. 4**c**, bottom; n = 29) during the choice epoch on easy (% Ipsi. odor = 5, 20, 80, or 95) and difficult (% Ipsi. odor = 40, 50, or 60) trials shown separately for ipsilateral (Ipsi.) and contralateral (Contra.) choices. Each neuron is represented by a connected pair of small symbols. Large symbols show population medians. Ipsi. choices, $p = 0.642$, two-tailed Mann-Whitney U test; Contra. choices, $p = 0.5963$, two-tailed Mann-Whitney U test.

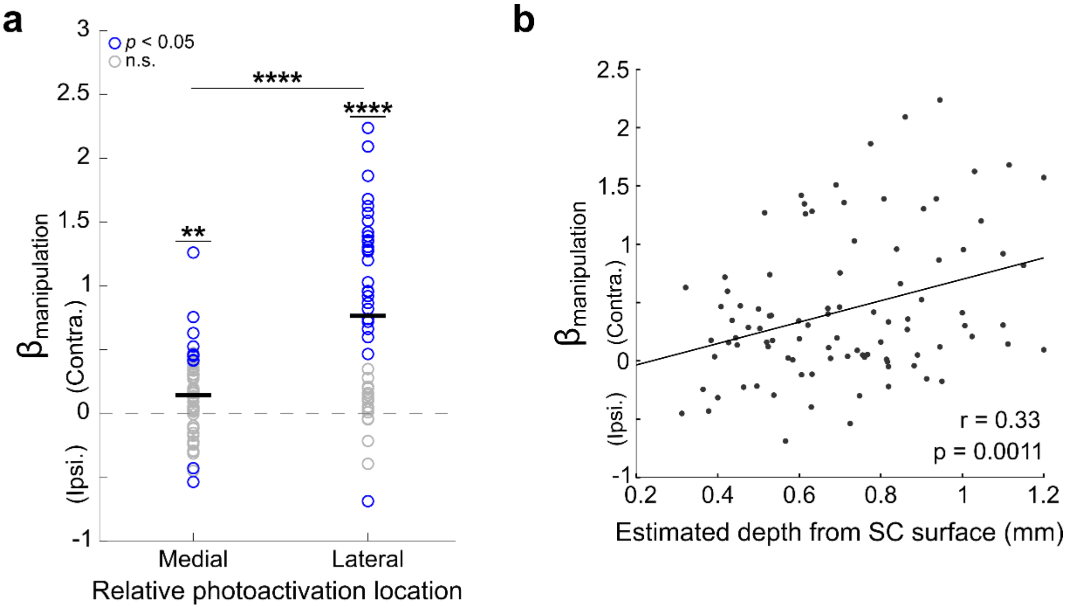

Supplementary Figure 9: Effect of mediolateral and dorsoventral photoactivation location on choice bias in Gad2-Cre mice.

(**a**) Photoactivation of ChR2+ GABAergic SC neurons in relatively medial (n= 52 sessions; 6 mice) and lateral (n = 44 sessions; 5 mice) SC. Horizontal lines show population means. **** $p < 0.0001$; ** $p < 0.01$, one-sample and two-sample t-tests. Mediolateral location was estimated based on histological reconstruction (Supplementary Fig. 1).

(**b**) Effect of intermediate and deep layer dorsoventral photoactivation location on choice bias (n = 96; $p = 0.0011$, Pearson correlation coefficient). Dorsoventral location was back-calculated for each session based on the ventral advancement of the optetrode between sessions (Methods) and histological reconstruction of the final optetrode depth from the surface of the superior colliculus (Supplementary Fig. 1).

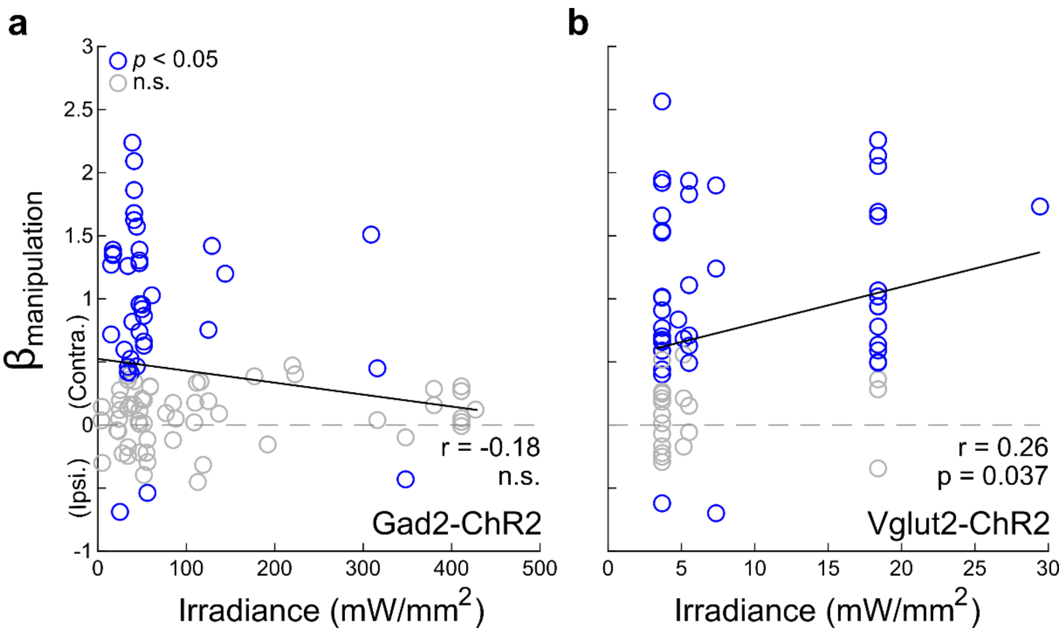

Supplementary Figure 10: Effect of light power of photoactivation on choice bias.
(**a**) Effect of light power for photoactivation of GABAergic SC neurons on choice bias (n = 96; p = 0.08, Pearson correlation coefficient).
(**b**) As in **a**, for photoactivation of glutamatergic SC neurons (n = 64; p = 0.037, Pearson correlation coefficient).

Supplementary Table 1: Numbers and types of sessions performed by, and numbers of neurons recorded from, each Gad2-Cre mouse.

Mouse	Stimulation/behavior (# of sessions)	Stimulation/recording (# of neurons)	Behavior/recording (# of neurons)	Stimulation/behavior/recording (# of neurons)
Abw25	12	12	12	—
Abw35	10	35	29	—
Abw36	7	—	—	—
Abw42	8	33	29	—
Abw43	8	31	16	—
Abw49	8	44	42	9
Abw53	10	38	33	12
Abw57	8	38	36	14
Abw61	8	29	21	16
Abw63	8	22	8	12
Abw67	9	19	13	2
Total	96 sessions	301 neurons	239 neurons	65 neurons

Supplementary Table 2: Firing rate change of individual neurons in response to light ($p < 0.05$, Mann-Whitney U tests) for stimulation/behavior/recording sessions.

	Light elicited firing rate change, Choice epoch (fraction of neurons)			Light elicited firing rate change, Post-choice epoch (fraction of neurons)		
Cell type	Light-excited	Light-inhibited	No change	Light-excited	Light-inhibited	No change
GABAergic	10/14	1/14	3/14	3/14	0/14	11/14
Unidentified	13/51	11/51	27/51	8/51	2/51	40/51

Supplementary Table 3: Light powers used for photoactivation ("stimulation/behavior", "stimulation/recording" and "stimulation/behavior/recording") sessions.

Mice	Power at fiber tip (mW)	Irradiance (mW/mm^2)	Number of sessions
Gad2-ChR2	0.12 –2.4	4.4 – 88	68
	3 - 6.1	109 – 223	15
	8.4 – 11.6	309 – 427.2	13
Gad2-YFP	7	257.6	13
	12	441.6	50
Vglut2-ChR2	0.1 – 0.8	3.7 – 29.4	64
Vglut2-YFP	1.5	55.2	30
	12	441.6	22
