# Supplementary material for: Inhibitory neurons in the superior colliculus mediate selection of spatially-directed movements
Source: Commun Biol. 2021 Jun 11;4:719. doi: 10.1038/s42003-021-02248-1 (PMC8196039; doi:10.1038/s42003-021-02248-1)
Supplement: Supplementary file 3 — Reporting Summary [file 42003_2021_2248_MOESM3_ESM.pdf]

nature research

Corresponding author(s): Gidon Felsen

Last updated by author(s): May 7, 2021

Reporting Summary

Nature Research wishes to improve the reproducibility of the work that we publish. This form provides structure for consistency and transparency in reporting. For further information on Nature Research policies, see Authors & Referees and the Editorial Policy Checklist .

Statistics

For all statistical analyses, confirm that the following items are present in the figure legend, table legend, main text, or Methods section.

n/a	Confirmed	
☐	☒	The exact sample size (n) for each experimental group/condition, given as a discrete number and unit of measurement
☐	☒	A statement on whether measurements were taken from distinct samples or whether the same sample was measured repeatedly
☐	☒	The statistical test(s) used AND whether they are one- or two-sided<br>*Only common tests should be described solely by name; describe more complex techniques in the Methods section.*
☒	☐	A description of all covariates tested
☐	☒	A description of any assumptions or corrections, such as tests of normality and adjustment for multiple comparisons
☐	☒	A full description of the statistical parameters including central tendency (e.g. means) or other basic estimates (e.g. regression coefficient) AND variation (e.g. standard deviation) or associated estimates of uncertainty (e.g. confidence intervals)
☐	☒	For null hypothesis testing, the test statistic (e.g. F, t, r) with confidence intervals, effect sizes, degrees of freedom and P value noted<br>*Give P values as exact values whenever suitable.*
☒	☐	For Bayesian analysis, information on the choice of priors and Markov chain Monte Carlo settings
☒	☐	For hierarchical and complex designs, identification of the appropriate level for tests and full reporting of outcomes
☐	☒	Estimates of effect sizes (e.g. Cohen's d, Pearson's r), indicating how they were calculated

Our web collection on statistics for biologists contains articles on many of the points above.

Software and code

Policy information about availability of computer code

Data collection	Behavior: controlled by MATLAB (2008b) and Linux real-time kernel. Recordings: Neuralynx data acquisition system and Cheetah software
Data analysis	Modeling and neural analysis: MATLAB(2015a/2019a), MClust 4.4.07. Behavioral analysis: Sci-Kit Learn Python package (version 0.21.3)

For manuscripts utilizing custom algorithms or software that are central to the research but not yet described in published literature, software must be made available to editors/reviewers. We strongly encourage code deposition in a community repository (e.g. GitHub). See the Nature Research guidelines for submitting code & software for further information.

Data

Policy information about availability of data

All manuscripts must include a data availability statement. This statement should provide the following information, where applicable:

- Accession codes, unique identifiers, or web links for publicly available datasets
- A list of figures that have associated raw data
- A description of any restrictions on data availability

The data that support the findings of this study and the custom MATLAB code are available from the corresponding author upon reasonable request.

Field-specific reporting

Please select the one below that is the best fit for your research. If you are not sure, read the appropriate sections before making your selection.

☒ Life sciences ☐ Behavioural & social sciences ☐ Ecological, evolutionary & environmental sciences

For a reference copy of the document with all sections, see nature.com/documents/nr-reporting-summary-flat.pdf

Life sciences study design

All studies must disclose on these points even when the disclosure is negative.

Sample size	Sample size was deemed adequate based on previous studies using similar techniques.
Data exclusions	Mice were excluded from analyses based on the misalignment of the optic fiber with ChR2 expression, lack of ChR2 expression, and misplacement of optetrode outside of the SC (13 mice were excluded from the final data set). Neurons were excluded from "choice epoch" analyses if firing rates during the epoch were below 2.5 spikes/s. Some analyses only included a subset of neurons; criteria for inclusion are outlined in the manuscript.
Replication	Findings from this study have not been replicated, although effects were consistent across mice.
Randomization	All mice were randomly assigned to experimental or control groups.
Blinding	Blinding was not implemented in this study.

Reporting for specific materials, systems and methods

We require information from authors about some types of materials, experimental systems and methods used in many studies. Here, indicate whether each material, system or method listed is relevant to your study. If you are not sure if a list item applies to your research, read the appropriate section before selecting a response.

Materials & experimental systems

n/a	Involved in the study	
☒	☐	Antibodies
☒	☐	Eukaryotic cell lines
☒	☐	Palaeontology
☐	☒	Animals and other organisms
☒	☐	Human research participants
☒	☐	Clinical data

Methods

n/a	Involved in the study	
☒	☐	ChIP-seq
☒	☐	Flow cytometry
☒	☐	MRI-based neuroimaging

Animals and other organisms

Policy information about studies involving animals; ARRIVE guidelines recommended for reporting animal research

Laboratory animals	All mice were obtained from Jackson Labs and were 6- 12 month old males. Strains used: wild-type C57BL/6J, heterozygous Gad2-ires-Cre (Gad2tm2(cre)Zjh/J). Homozygous Vglut2-ires-Cre (Slc17a6tm2(cre)Lowl/J).
Wild animals	NA
Field-collected samples	NA
Ethics oversight	All procedures were approved by University of Colorado School of Medicine Institutional Animal Care and Use Committee.

Note that full information on the approval of the study protocol must also be provided in the manuscript.
